# 5-Aminolevulinic Acid Drives Coordinated Astaxanthin and Lipid Accumulation in Green Alga *Chromochloris zofingiensis*

**DOI:** 10.3390/foods15101768

**Published:** 2026-05-17

**Authors:** Jinrui Gao, Zhongliang Sun, Bin Liu, Yu Zhang, Liqin Sun

**Affiliations:** Yantai Key Laboratory of Characteristic Agricultural Bioresource Conservation & Germplasm Innovative Utilization, School of Life Sciences, Yantai University, Yantai 264000, China; gaojinrui@s.ytu.edu.cn (J.G.); zlsun@ytu.edu.cn (Z.S.); liubin@ytu.edu.cn (B.L.)

**Keywords:** *Chromochloris zofingiensis*, 5-aminolevulinic acid (5-ALA), astaxanthin, lipid, transcriptomics

## Abstract

*Chromochloris zofingiensis*, a photosynthetic microalga, has attracted considerable attention due to its ability to simultaneously accumulate lipids and astaxanthin. However, the induction of lipid and secondary metabolite biosynthesis by abiotic stress is typically accompanied by growth inhibition, resulting in a trade-off between metabolite accumulation and biomass production. In recent years, phytohormones have emerged as an effective strategy for regulating microalgal metabolism, owing to their high specificity and low effective dosage. In this study, 5-aminolevulinic acid (5-ALA) was applied under nitrogen-deficient conditions, and its effects on growth, photosynthesis, lipid metabolism, and carotenoid biosynthesis were systematically evaluated through integrated physiological, biochemical, and transcriptomic analyses. The results showed that 5-ALA had no significant effect on biomass accumulation or photosynthetic performance. However, at 2 μM, 5-ALA exhibited the strongest promotive effect on lipid and astaxanthin accumulation, with total fatty acids (TFA) and triacylglycerol (TAG) contents increasing by 13.3% and 25.7%, respectively, and total carotenoids and astaxanthin contents increasing by 15.6% and 17.2%, respectively. Under semi-continuous cultivation, TAG and astaxanthin productivities were enhanced by 13.9% and 22.9%, reaching 164 mg L^−1^ d^−1^ and 2.15 mg L^−1^ d^−1^, respectively. Transcriptomic analysis revealed that 5-ALA induced only limited transcriptional changes but enhanced glycolysis, central carbon metabolism, and nitrogen recycling, thereby increasing the supply of carbon precursors and energy. Notably, no significant transcriptional changes were observed in the carotenoid biosynthesis pathway, indicating that the enhanced accumulation of total carotenoids and astaxanthin was likely driven by increased metabolic flux. In terms of lipid metabolism, the upregulation of pathways involved in the conversion of membrane lipids into TAG, together with the downregulation of TAG degradation pathways and enhanced carbon flux, collectively promoted TAG accumulation. Overall, this study demonstrates that supplementation with 2 μM 5-ALA provides a practical and cost-effective strategy for the efficient co-production of lipids and astaxanthin in *C. zofingiensis*.

## 1. Introduction

Microalgae have emerged as promising cell factories for the production of functional foods, biofuels, and feed additives due to their high photosynthetic efficiency, rapid growth rate, and strong capacity for metabolite synthesis [1,2]. Among them, *Chromochloris zofingiensis* has attracted increasing attention as an industrially relevant species because of its ability to simultaneously accumulate lipids and high-value carotenoids, particularly astaxanthin [3]. The lipid fraction of this alga is enriched in fatty acids such as palmitic acid (C16:0) and oleic acid (C18:1), which are suitable for biodiesel production [4,5], while also containing polyunsaturated fatty acids with recognized health benefits [6]. Astaxanthin, a potent antioxidant, exhibits substantially higher free radical scavenging activity than β-carotene and vitamin E, making it valuable for pharmaceutical and nutraceutical applications [7]. However, the accumulation of lipids and astaxanthin typically relies on abiotic stress induction (e.g., nitrogen deficiency), which often suppresses cell growth, thereby leading to a pronounced trade-off between biomass accumulation and metabolite synthesis [8,9]. Therefore, effectively alleviating or overcoming this trade-off has become a key research focus. To date, numerous studies have aimed to enhance astaxanthin and lipid production in *C. zofingiensis*. In general, the main strategies include optimization of culture conditions [10] and the development of multi-stage cultivation processes [10,11]. Nevertheless, these approaches often suffer from limited efficiency and increased operational costs, highlighting the need for more efficient and cost-effective regulatory strategies.

Phytohormones, as a class of chemical inducers, have recently shown great potential in regulating microalgal growth and metabolism, with expanding applications [12]. Compared with conventional environmental stress strategies, phytohormones exhibit higher specificity and require lower effective dosages, and they can simultaneously promote cell proliferation and metabolite accumulation, enhance growth alone, or increase product accumulation without markedly inhibiting growth [13,14]. Therefore, they are considered a promising approach to mitigate the inherent trade-off between growth and product synthesis. 5-Aminolevulinic acid (5-ALA), a key precursor in the biosynthesis of chlorophyll, heme, and other tetrapyrrole compounds, is widely distributed in living organisms [15]. In higher plants, 5-ALA functions as a growth regulator that enhances photosynthetic efficiency, improves stress tolerance, and modulates carotenoid metabolism [16,17]. Its physiological effects are concentration-dependent, exhibiting a typical hormetic response characterized by stimulation at low doses and inhibition at high doses [18]. In terms of mechanisms, as a precursor of chlorophyll biosynthesis, exogenous 5-ALA application can enhance photosynthetic performance and thereby influence cellular energy metabolism, which is closely associated with biomass accumulation as well as lipid and carotenoid biosynthesis through the supply of NADPH and ATP [19]. In addition, its downstream metabolites, such as heme, serve as essential cofactors in mitochondrial electron transport and antioxidant systems, suggesting a potential role in regulating cellular redox balance, secondary metabolism, and stress tolerance [17,20]. Increasing evidence suggests that 5-ALA also regulates growth and metabolic processes in microalgae. For example, in the green microalga *Haematococcus pluvialis*, 5-ALA enhances biomass production by promoting photosynthetic CO_2_ assimilation [21], whereas in the red alga *Porphyridium purpureum,* it stimulates arachidonic acid biosynthesis [22]. These findings indicate that the effects of 5-ALA are species-specific, with regulatory roles that may vary considerably among different microalgae. However, to date, the application of 5-ALA in microalgae remains largely unexplored, and the underlying mechanisms governing its regulation of microalgal growth and metabolism are still not well understood. Particularly in the unicellular photosynthetic green alga *C. zofingiensis*—an ideal producer of lipids and astaxanthin and a valuable model for studying lipid metabolism and astaxanthin biosynthesis—the effects of 5-ALA remain largely unexplored and merit systematic investigation. To date, only a few studies have examined the influence of phytohormones on *C. zofingiensis*, such as rutin [13,23].

In this study, *C. zofingiensis* was employed as a model organism to systematically investigate the dose-dependent effects of 5-ALA on growth, photosynthetic performance, lipid metabolism, and pigment accumulation under nitrogen-deficient conditions. By integrating physiological, biochemical, and transcriptomic analyses, we aimed to elucidate the multi-level regulatory mechanisms underlying 5-ALA-mediated metabolic reprogramming. Furthermore, the potential of 5-ALA to enhance fatty acid and astaxanthin productivity was evaluated under semi-continuous cultivation. This study provides new insights into hormone-mediated metabolic regulation and offers a feasible strategy for overcoming the trade-off between growth and product synthesis in microalgae.

## 2. Material and Methods

### 2.1. Materials

*Chromochloris zofingiensis* (ATCC 30412) was obtained from the American Type Culture Collection (ATCC) (Manassas, VA, USA). 5-Aminolevulinic acid (5-ALA, 99% purity, Lot No: OK20231025U) was purchased from Beijing Wokai Biotechnology Co., Ltd. (Beijing, China). Microalgae were cultured in Kuhl freshwater medium. The composition of the medium was as follows (per liter): 1.01 g KNO_3_; 0.62 g NaH_2_PO_4_·H_2_O; 0.089 g Na_2_HPO_4_·2H_2_O; 0.247 g MgSO_4_·7H_2_O; 14.7 mg CaCl_2_·2H_2_O; 6.95 mg FeSO_4_·7H_2_O; 0.061 mg H_3_BO_3_; 0.169 mg MnSO_4_·H_2_O; 0.287 mg ZnSO_4_·7H_2_O; 0.0025 mg CuSO_4_·5H_2_O; and 0.01235 mg (NH_4_)_6_Mo_7_O_24_·4H_2_O. The pH of the medium was adjusted to 6.5 prior to sterilization.

### 2.2. Cultivation Conditions

After activation, the algal cells were inoculated into a T8 double-sided, double-layer photobioreactor with an initial OD_750_ of 1.5 and cultivated under nitrogen-deficient conditions (Kuhl medium without KNO_3_). The cultures were maintained at a light intensity of 80 μE·m^−2^·s^−1^ under continuous illumination (24 h), with continuous aeration supplied by 1.5% CO_2_, at 25 °C. All experiments were conducted with three biological replicates. For batch culture, a 5-ALA concentration gradient of 0 (control), 1, 2, 4, and 6 μM was established, and the microalgae were cultured for 96 h. For semi-continuous cultivation, experiments were conducted under the same conditions for a total of 6 days. After 3 days of cultivation, 30% of the culture was harvested by centrifugation replaced with an equal volume of fresh nitrogen-deficient medium supplemented with the corresponding concentration of 5-ALA. This replacement procedure was performed daily (dilution rate: 0.3 d^−1^) for three cycles.

### 2.3. Determination of Growth Characteristics

Biomass was expressed as cell dry weight. A 3 mL algal culture was collected by centrifugation, washed twice with ultrapure water, and filtered through a GF/C membrane (1.2 µm pore size), followed by vacuum drying at 80 °C to constant weight. The dry weight was calculated based on the difference in membrane mass before and after filtration. Cell density was determined using a hemocytometer under an optical microscope. The total cell number in 1 mL of sample was calculated according to the following equation: N = A/5 × 25 × 10^4^ × B, where A is the total number of cells counted in five central grids, and B is the dilution factor. The optical density at 750 nm (OD_750_) was measured using a NanoDrop microvolume UV–Vis spectrophotometer.

### 2.4. Analysis of Photosynthetic Characteristics

Chlorophyll fluorescence parameters, including the maximum quantum yield of photosystem II (Fv/Fm), electron transport rate (ETR), and non-photochemical quenching (NPQ), were measured using a chlorophyll fluorometer (PAM-CONTROL, Walz, Germany) according to the method described previously [24]. In brief, algal samples were dark-adapted for 15 min prior to measurement and then transferred into measuring cuvettes. A saturating pulse of actinic light was applied to determine the maximum fluorescence yield of dark-adapted cells (Fm) and light-adapted cells (Fm′). Fv/Fm was calculated as (Fm − F0)/Fm, where F0 represents the minimum fluorescence yield in the dark-adapted state. ETR and NPQ were automatically calculated by the instrument software, with NPQ determined according to the formula (Fm − Fm′)/Fm′. All measurements were performed at room temperature under dark conditions with continuous stirring to prevent cell sedimentation and minimize interference.

### 2.5. Fatty Acid Extraction and Quantification

Lipid extraction and analysis were performed according to the method described previously [25]. In brief, freeze-dried algal biomass was disrupted using a tissue grinder (Qiagen, TissueLyser II, Hilden, Germany), and total lipids were extracted using a methanol–chloroform system. Absolute methanol containing 0.05% BHT and chloroform were added at a volume ratio of 1:2, followed by vortex extraction for 15 min. Subsequently, 1/4 volume of 0.75% NaCl aqueous solution was added, and the mixture was thoroughly vortexed, allowed to stand for 5 min, and centrifuged at 3000 rpm for 10 min. The chloroform phase containing lipids and pigments was collected and dried under nitrogen gas to complete lipid and pigment extraction. The extracted lipids were separated by thin-layer chromatography (TLC) into neutral lipids (TAG), diacylglycerols (DAG), free fatty acids (FFA), and polar lipids (PL) by using a developing solvent consisting of n-hexane: methyl tert-butyl ether: acetic acid (8:2:0.2, *v*/*v*/*v*). The target bands were scraped and subjected to transmethylation using 1% (*v*/*v*) sulfuric acid in methanol at 85 °C for 2.5 h, with heptadecanoic acid (C17:0) as an internal standard. The resulting fatty acid methyl esters (FAMEs) were extracted with n-hexane and analyzed by gas chromatography (7890B GC system, Agilent Technologies, Santa Clara, CA, USA) equipped with a DB-WAX capillary column (Agilent Technologies, Santa Clara, CA, USA) to determine fatty acid composition and content according to the method described previously [25].

### 2.6. Pigment Extraction and Quantification

Pigment extracts and analysis were performed according to the method described previously [25]. In brief, Nitrogen-dried lipid extracts (including pigments) were re-dissolved in acetone and centrifuged, and the supernatant was subjected to high-performance liquid chromatography (HPLC, Waters, e2695, Milford, MA, USA) for separation and quantification. Most pigments were separated using an OSAKA SODA CAPCELL PAK C18 column (4.6 mm × 250 mm) (Osaka, Japan), whereas lutein and zeaxanthin were separately resolved using a Waters SPHERISORB C18 ODS1 (Milford, MA, USA) column (4.6 mm × 250 mm). Mobile phase A consisted of methanol: ethyl acetate (68:32, *v*/*v*), while mobile phase B consisted of acetonitrile: methanol: 0.1 M Tris-HCl (84:2:14, *v*/*v*/*v*). The elution program was as follows: 100% mobile phase B was linearly changed to 100% mobile phase A over 10 min, followed by holding at 100% mobile phase A for 15 min. Subsequently, the system was linearly returned to 100% mobile phase B within 3 min and maintained for an additional 5 min. The flow rate was set at 1.2 mL/min. Pigment contents were quantified using the external standard calibration method.

### 2.7. Transcriptomic Analysis

Samples were collected at 6 h, 12 h, and 24 h, immediately frozen in liquid nitrogen, and stored at −80 °C until further analysis. RNA extraction was performed using the TaKaRa MiniBEST Plant RNA Extraction Kit (Cat. No. 9769A, Takara Bio Inc., Shiga, Japan) according to the manufacturer’s instructions. RNA library construction was performed using the TruSeq RNA Sample Preparation Kit (Cat. No. RS-122-2002, Illumina, San Diego, CA, USA) according to the manufacturer’s instructions. Sequencing was conducted on the Illumina HiSeq X Ten/NovaSeq 6000 platform by Majorbio Bio-Pharm Technology Co., Ltd. (Shanghai, China), with a paired-end read length of 150 bp [26]. The clean reads were aligned to the genome of *C. zofingiensis* (https://phytozome-next.jgi.doe.gov/info/Czofingiensis_v5_2_3_2; accessed on 26 March 2026) with the software TopHat (version 2.0.4). The gene transcriptional abundance was expressed as fragments per kilobase million (FPKM). Differentially expressed genes (DEGs) were identified using the DESeq2 package with the following criteria: |log_2_ fold change (FC)| ≥ 1, adjusted *p*-value < 0.05, and fragments per kilobase of transcript per million mapped reads (FPKM) > 1. The control for each comparison was the corresponding culture at the same time point without 5-ALA treatment under nitrogen-deficient conditions.

### 2.8. Statistics and Data Analysis

All experiments were performed with three biological replicates, and the data are presented as mean ± standard deviation (SD). Statistical significance among groups was evaluated using one-way analysis of variance (ANOVA) followed by Dunnett’s multiple comparison test to compare treatment groups with the control group. Differences were considered statistically significant at *p* < 0.05.

## 3. Results and Discussion

### 3.1. Effects of 5-ALA on the Growth of Chromochloris zofingiensis

**Figure 1:**
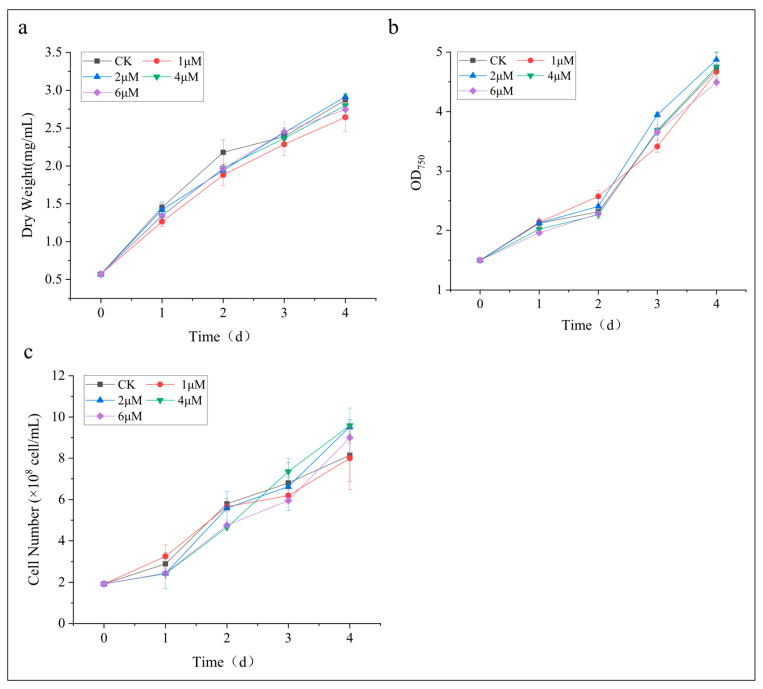
Effects of different concentrations of 5-ALA on the growth of *Chromochloris zofingiensis*. (**a**) Dry weight; (**b**) OD_750_; (**c**) Cell density.

To investigate the influence of 5-ALA on biomass accumulation in *C. zofingiensis*, different concentration gradients of 5-ALA were applied in this study under nitrogen-deficient conditions. The growth trends of all 5-ALA-treated groups (1, 2, 4, and 6 μM) were generally consistent with that of the control, showing a continuous increase over the cultivation period (Figure 1). During the 4-day cultivation, the control group and all 5-ALA-treated groups exhibited similar dynamic patterns in dry weight (Figure 1a), OD_750_ (Figure 1b), and cell density (Figure 1c), suggesting that exogenous 5-ALA did not significantly alter the basic growth characteristics of *C. zofingiensis* under nitrogen-deficient conditions, where cell growth was inherently constrained. Statistical analysis showed that there were no significant differences in biomass-related parameters between the treated groups and the control at any time point (*p* > 0.05). At day 4, the dry weight of the control reached 2.88 mg/mL, while those of the 1, 2, 4, and 6 μM treatments were 2.65, 2.90, 2.78, and 2.70 mg/mL, respectively. The corresponding OD_750_ values were 2.40, 2.51, 2.37, 2.45, and 2.35, and the cell densities were 9.8 × 10^8^, 9.2 × 10^8^, 9.5 × 10^8^, 10.1 × 10^8^, and 8.9 × 10^8^ cells/mL, respectively. Although minor fluctuations were observed among treatments, none of these differences were statistically significant.

Overall, these results indicate that 5-ALA had no significant effect on biomass accumulation of *C. zofingiensis* within the range of 1–6 μM under nitrogen-deficient conditions. This finding differs from previous studies reporting that exogenous 5-ALA supplementation at appropriate concentrations significantly promoted biomass accumulation in *Porphyridium purpureum* [22] and *Phaeodactylum tricornutum* [27]. However, it should be noted that these studies were conducted under growth-favorable conditions for microalgae, rather than under abiotic stress conditions such as nitrogen deficiency employed in the present study. Similarly, Li et al. reported in *H*. *pluvialis* that exogenous 5-ALA applied under favorable growth conditions promoted biomass accumulation by enhancing Rubisco activity (4 μM 5-ALA, increasing biomass by 23.8%). In contrast, the addition of exogenous 5-ALA during the high-light induction stage failed to improve biomass accumulation [21]. Therefore, the lack of a growth-promoting effect observed in this study is likely attributable to the nitrogen-deficient conditions employed in this study, under which cell growth is primarily constrained by nitrogen availability rather than photosynthetic carbon assimilation [8]. In addition, nitrogen deficiency often imposes inhibitory effects on the photosynthetic system [24,28], which may further weaken the growth-promoting effect of 5-ALA. Consequently, under nitrogen-deficient conditions (which are known to promote lipid and astaxanthin accumulation), exogenous 5-ALA addition in *C. zofingiensis* did not translate into a significant increase in biomass, nor did it exert a noticeable inhibitory effect on cell growth.

It is important to note that the cultures appeared to remain in the exponential growth phase rather than reaching the stationary phase by day 4; lipid and secondary carotenoid accumulation may further increase when cultures enter the stationary phase. However, considering that TFA and astaxanthin contents had already reached relatively high levels [9], and significant differences were observed between the control and treatment groups, particularly in the 2 μM 5-ALA treatment (See Section 3.3 and Section 3.4), a cultivation period of 4 days was employed in this study. Nevertheless, this represents a methodological limitation of the present study. Extending the cultivation period in future studies may provide additional insights into the long-term effects of 5-ALA and the potential maximum accumulation of lipids and astaxanthin.

### 3.2. Effects of 5-ALA on Photosynthesis in Chromochloris zofingiensis

**Figure 2:**
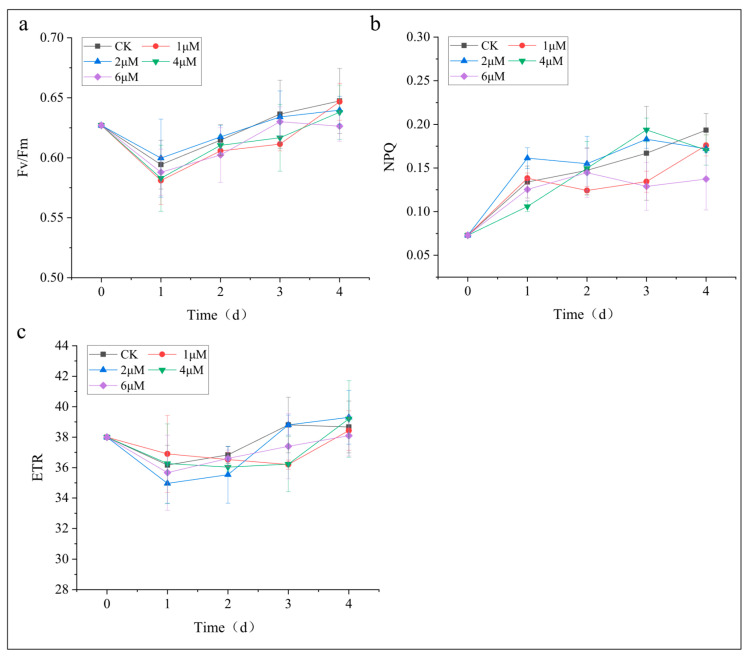
Effects of different concentrations of 5-ALA on photosynthesis in *Chromochloris zofingiensis*. (**a**) Maximum quantum yield of PSII (Fv/Fm); (**b**) Non-photochemical quenching (NPQ); (**c**) Electron transport rate (ETR).

5-ALA is known to participate in tetrapyrrole biosynthesis and has the potential to regulate photosynthetic processes [20,29]. Therefore, in this study, photosynthetic performance was systematically evaluated to determine whether exogenous 5-ALA affects the photosynthetic activity of *C. zofingiensis* by assessing key photosynthetic parameters, including Fv/Fm, ETR and NPQ. As shown in Figure 2a, the Fv/Fm values exhibited a slight decrease on day 1, followed by a gradual recovery to stable levels across all treatments, with no significant differences observed between the control and 5-ALA-treated groups, ranging from 0.63 to 0.65 (Figure 2a). Regarding NPQ levels, both the control and 5-ALA-treated groups exhibited an increasing trend on day 1, which may be attributed to the activation of stress responses under nitrogen-deficient conditions. Thereafter, NPQ levels in all groups fluctuated within a certain range. No significant differences were observed between the control and the treated groups at any time point (Figure 2b). The dynamic changes in ETR closely paralleled those of Fv/Fm. Similarly, no significant differences in ETR were observed between the control and the 5-ALA-treated groups (Figure 2c). Collectively, these results suggest that 5-ALA did not significantly affect the overall functionality of the photosynthetic apparatus within the concentration range of 1–6 μM.

To our knowledge, no previous study has reported the effects of 5-ALA on the photosynthetic performance of *C. zofingiensis*. Studies in other microalgal species have shown that 5-ALA may enhance carbon assimilation and improve photosynthetic performance [21]; however, these effects were generally observed under favorable growth conditions where the photosynthetic system remained relatively stable. In contrast, the present study was conducted under nitrogen-deficient conditions, an abiotic stress known to impose inhibitory effects on the photosynthetic machinery of microalgae [30], which may not be effectively alleviated by exogenous 5-ALA supplementation. Moreover, previous studies have suggested that the effects of phytohormones on photosynthetic performance are often consistent with their effects on growth [13,31,32]. In the present study, the absence of significant changes in photosynthetic performance was consistent with the lack of significant biomass enhancement following 5-ALA treatment, suggesting that exogenous 5-ALA did not markedly alter the physiological status of *C. zofingiensis* under nitrogen-deficient conditions.

### 3.3. Effects of 5-ALA on Lipid Metabolism of Chromochloris zofingiensis

**Figure 3:**
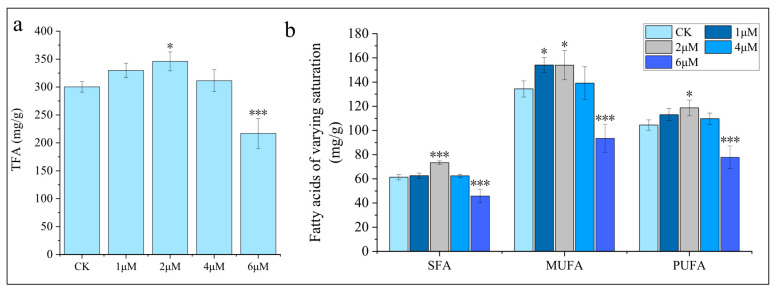
Effects of 5-ALA on lipid metabolism in *Chromochloris zofingiensis*. (**a**) Total fatty acid (TFA) content; (**b**) TFA distribution by saturation degree. Asterisks indicate significant differences compared with the control (* *p* < 0.05, *** *p* < 0.001).

During the induction process, varying concentrations of 5-ALA exhibited differential effects on lipid biosynthesis in *C. zofingiensis* (Figure 3). For total fatty acids (TFA), lower concentrations of 5-ALA promoted lipid accumulation, whereas higher concentrations exerted an inhibitory effect, which is consistent with the concentration-dependent characteristics commonly observed for plant growth regulators [33,34]. Specifically, the 2 μM treatment resulted in a significant increase of 13.3% compared with the control group (*p* < 0.05), reaching 346 mg/g (Figure 3a). By comparison, under optimal growth conditions, the TFA content is typically only around 100 mg/g [3]. The fatty acid composition remained unchanged, with all treatment groups predominantly composed of C18:1, C18:2, C16:0, and C18:3 (Table 1). Instead, a general and coordinated increase was observed, with saturated fatty acids (SFA), monounsaturated fatty acids (MUFA), and polyunsaturated fatty acids (PUFA) increasing by 25.0%, 14.8%, and 9.5%, respectively (Figure 3b). In contrast, a high concentration (6 μM) significantly inhibited both individual fatty acid components and the overall TFA content (decreased by 28.3%, *p* < 0.001). Similarly, no structural changes in fatty acid composition were observed under this condition; instead, a coordinated decrease occurred, with SFA, MUFA, and PUFA all significantly reduced. Treatments with 1 μM and 4 μM 5-ALA did not significantly affect TFA levels, nor did they cause significant changes in fatty acid composition or in fatty acid classes with different degrees of saturation.

TAG, as the major storage lipid component [35], exhibited a variation trend consistent with that of TFA, but with a more pronounced response (Figure S1). Treatments with 1, 2, and 4 μM 5-ALA all promoted TAG accumulation, with the highest level observed at 2 μM, showing a 25.7% increase compared with the control (*p* < 0.001), followed by 4 μM (24.6%, *p* < 0.001) and 1 μM (9.8%, *p* < 0.05). The 2 μM 5-ALA treatment significantly promoted the accumulation of major fatty acid species in TAG, including C16:0, C18:1, and C18:2, which increased by 27.3%, 19.3%, and 38.9%, respectively (Table S1). Meanwhile, SFA, MUFA, and PUFA all showed significant increases, among which PUFA exhibited the highest increase of 47.1%. Under 6 μM, the promoting effect was attenuated but no significant inhibition was observed, indicating that TAG biosynthesis exhibits a certain tolerance to high concentrations of 5-ALA.

In contrast to storage lipids, phospholipids (PL) exhibited a relatively stable response to 5-ALA. Only the 1 and 2 μM treatments showed a significant promotive effect, with the magnitude of increase being lower than that observed for the corresponding TAG. The 2 μM treatment exhibited the strongest promotion, increasing by 19.4% compared with the control (*p* < 0.01), followed by the 1 μM treatment, which increased by 10.3% (*p* < 0.05). Similarly, the changes in fatty acid composition (Table S2), as well as in the contents of SFA, MUFA, and PUFA, were relatively modest (Figure S1). Under abiotic stress conditions such as nitrogen deprivation, a conversion from polar lipids to storage lipids often occurs, which may account for the more pronounced increase in TAG compared with the relatively minor changes in polar lipids [36,37,38].

Phytohormones have been widely reported to promote lipid accumulation in microalgae and generally exhibit a concentration-dependent effect [39,40,41]. However, studies focusing on the effects of phytohormones on lipid accumulation in *C*. *zofingiensis*, as well as the role of 5-ALA in regulating microalgal lipid metabolism, remain limited. Previous studies have shown that indole-3-acetic acid (IAA) can effectively enhance lipid accumulation in *C. zofingiensis* [23]. In addition, appropriate concentrations of 5-ALA were reported to promote lipid accumulation in *P*. *tricornutum* [27], whereas excessively high concentrations inhibited lipid accumulation in *P*. *purpureum* [22]. Consistent with most previous studies on hormone-mediated lipid regulation in microalgae, our results demonstrated that, within the tested concentration range, exogenous 5-ALA exhibited a typical concentration-dependent effect on lipid metabolism, with low concentrations promoting lipid biosynthesis and high concentrations inhibiting it. Notably, although previous studies suggested that 5-ALA may specifically stimulate the accumulation of certain fatty acids, such as arachidonic acid [22], no specific induction effect of 5-ALA on individual fatty acids or particular fatty acid classes was observed in the present study. In summary, among the tested conditions, 2 μM was identified as the optimal stimulatory concentration, under which TFA significantly increased by 13.3%, while TAG showed a 25.7% increase compared with the control group.

### 3.4. Effects of 5-ALA on Pigment Metabolism of Chromochloris zofingiensis

**Figure 4:**
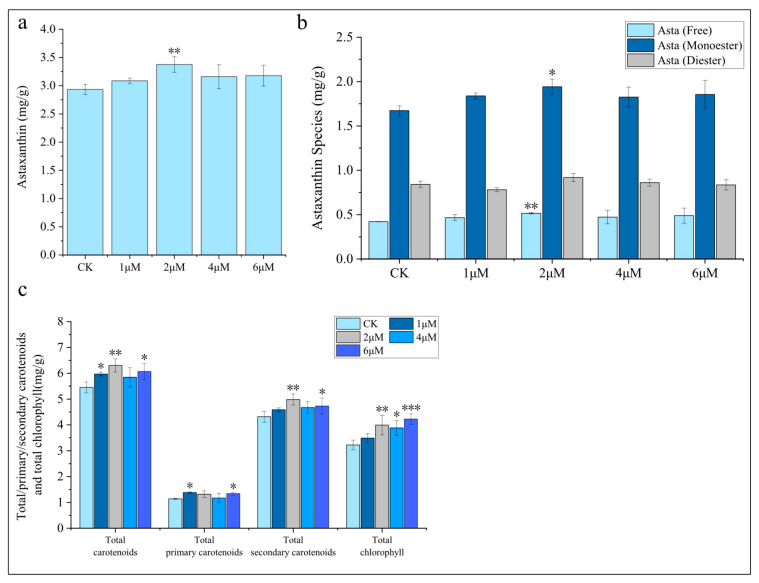
Effects of 5-ALA on pigment content in *Chromochloris zofingiensis*. (**a**) Total astaxanthin content; (**b**) Content of different astaxanthin species; (**c**) Total carotenoids, primary carotenoids, secondary carotenoids, and chlorophyll content. Asterisks indicate significant differences compared with the control (* *p* < 0.05, ** *p* < 0.01, *** *p* < 0.001).

This study systematically investigated the regulatory effects of exogenous 5-ALA on carotenoid and chlorophyll biosynthesis (Figure 4 and Figure S2). Astaxanthin is the most potent ketocarotenoid in terms of antioxidant activity, exhibiting an antioxidant capacity more than 500 times that of vitamin E [42]. It is a high-value bioactive compound derived from microalgae, with substantial market demand in the biopharmaceutical, functional food, and cosmetic industries [43,44,45,46,47]. The results indicated that 5-ALA can promote the accumulation of astaxanthin. Notably, the 2 μM 5-ALA treatment significantly increased astaxanthin content by 17.2% (*p* < 0.01), whereas other concentrations did not exhibit a significant promotive effect (Figure 4a). Natural astaxanthin exists in both free and esterified forms, with the esterified form further classified into astaxanthin monoesters and diesters. Studies have shown that esterified astaxanthin exhibits greater stability and stronger antioxidant capacity than free astaxanthin [48,49]. In *C. zofingiensis*, astaxanthin predominantly exists in esterified forms. 5-ALA treatment resulted in a 16.1% increase in astaxanthin monoesters (*p* < 0.05) and a 22.3% increase in astaxanthin diesters (*p* < 0.01) (Figure 4b). The promotive effect of 5-ALA on lipid biosynthesis may provide an increased supply of free fatty acids for astaxanthin esterification (Figure 3).

In addition to astaxanthin, *C. zofingiensis* contains primary carotenoids such as β-carotene, zeaxanthin, neoxanthin, violaxanthin, and lutein, as well as secondary carotenoids including echinenone, canthaxanthin, adonixanthin, and ketolutein. Exogenous application of 5-ALA increased the total carotenoid content, with the most pronounced effect observed at 2 μM (increased by 15.6%), followed by 6 μM and 1 μM (Figure 4c). Notably, 2 μM 5-ALA did not enhance the levels of primary carotenoids but significantly promoted the accumulation of secondary carotenoids (15.5%), mainly astaxanthin and ketolutein (Figure S2).

As 5-ALA is a key precursor in chlorophyll biosynthesis, the changes in chlorophyll content were further analyzed. The results showed that 2, 4, and 6 μM 5-ALA all promoted an increase in total chlorophyll content (Figure 4c). Specifically, chlorophyll *a* levels were enhanced by 2, 4, and 6 μM 5-ALA, while chlorophyll *b* content was increased under 2 and 6 μM treatments (Figure S2).

In summary, exogenous 5-ALA promotes the accumulation of total carotenoids, astaxanthin, and chlorophyll. Among the tested concentrations, 2 μM 5-ALA exhibited the most pronounced effect on total carotenoid and astaxanthin accumulation. This response is markedly different from that observed in *H*. *pluvialis*, the only microalgal species currently used for the commercial production of astaxanthin, in which exogenous 5-ALA does not enhance astaxanthin accumulation [21]. Although both species are green microalgae, they employ distinct biosynthetic pathways and regulatory mechanisms for astaxanthin production, which may account for their differential responses to 5-ALA [25].

### 3.5. Transcriptomic Analysis Reveals the Molecular Mechanisms of 5-ALA Mediated Regulation in Chromochloris zofingiensis

**Figure 5:**
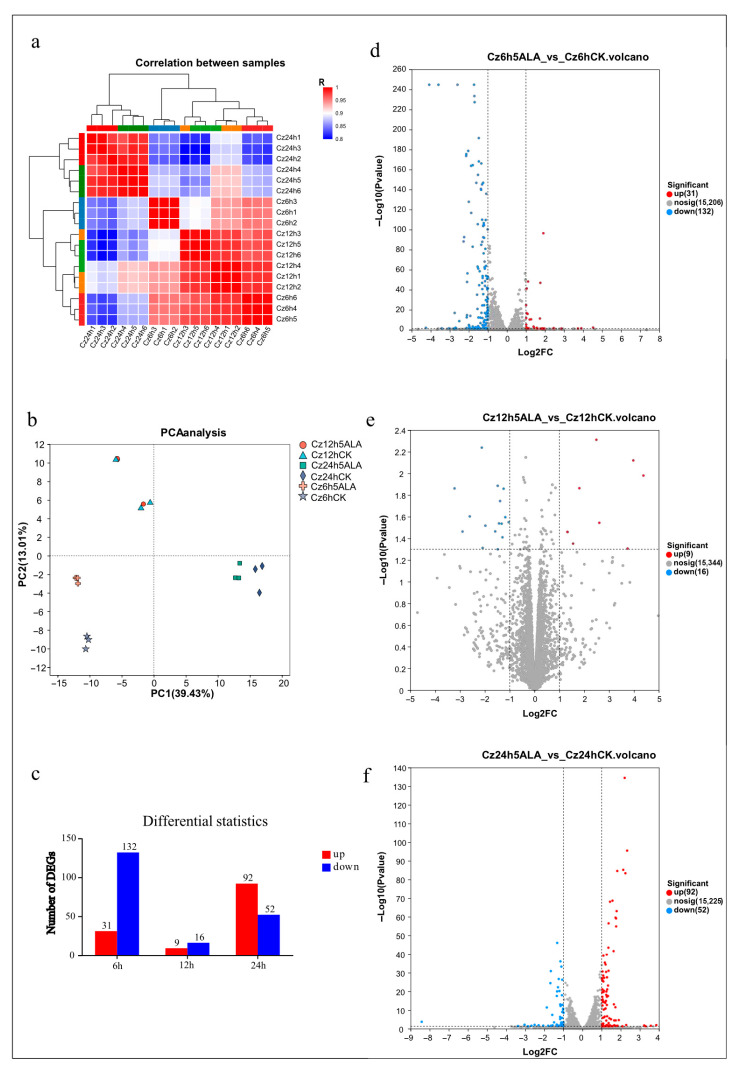
Global analysis of transcriptomes and DEGs. (**a**) Pearson correlation analysis among 5-ALA-treated samples based on global gene expression profiles; (**b**) Principal component analysis (PCA) of 5-ALA-treated samples based on global gene expression profiles; (**c**) KEGG pathway enrichment analysis of differentially expressed genes in 5-ALA-treated groups; (**d**–**f**) Volcano plots of DEGs at 6 h, 12 h, and 24 h after 5-ALA treatment. Red dots indicate significantly upregulated genes, blue dots indicate significantly downregulated genes, and gray dots indicate genes with no significant difference.

To verify the reliability of the transcriptome sequencing data and assess the overall variation among samples under different treatments, correlation analysis and principal component analysis (PCA) were conducted. The sample correlation heatmap (Figure 5a) revealed a clear separation between the 5-ALA treatment groups and the control group. The correlation coefficients (R^2^) among biological replicates at the same time point all exceeded 0.95, indicating strong reproducibility and robustness of the dataset. Hierarchical clustering analysis further showed that the 24 h treatment group formed a distinct branch, clearly separated from the 6 h, 12 h, and control groups. In contrast, the 6 h and 12 h treatment groups clustered together and were collectively distinct from the control, suggesting a time-dependent accumulation of transcriptional changes. In the heatmap, high correlations were primarily concentrated along the diagonal, whereas a markedly lower correlation was observed between the control and the 24 h treatment group, indicating that 5-ALA induced substantial time-dependent transcriptional reprogramming. The PCA results (Figure 5b) were consistent with the correlation analysis. The first two principal components (PC1 and PC2) explained 52.44% of the total variance, with PC1 (39.43%) mainly distinguishing treated samples from the control and PC2 (13.01%) reflecting temporal effects. The control group was located on the negative axis of PC1, whereas the 24 h treatment group was positioned on the positive axis and exhibited the greatest separation, indicating the most pronounced transcriptomic changes. The 6 h and 12 h groups were distributed in the intermediate region and followed a temporal trajectory along PC2.

Subsequently, differentially expressed genes (DEGs) in the 5-ALA treatment group at 6 h, 12 h, and 24 h were systematically analyzed (Figure 5c), and volcano plots (Figure 5d–f) were used to illustrate the overall distribution patterns of gene expression changes. The results showed that a total of 163 genes were significantly altered at 6 h, with downregulated genes predominating (132 genes). At 12 h, the number of DEGs markedly decreased to 25, with a nearly balanced proportion of upregulated and downregulated genes and a relatively dispersed distribution. By 24 h, the number of DEGs significantly increased to 144, with upregulated genes becoming dominant (92 genes).

**Figure 6:**
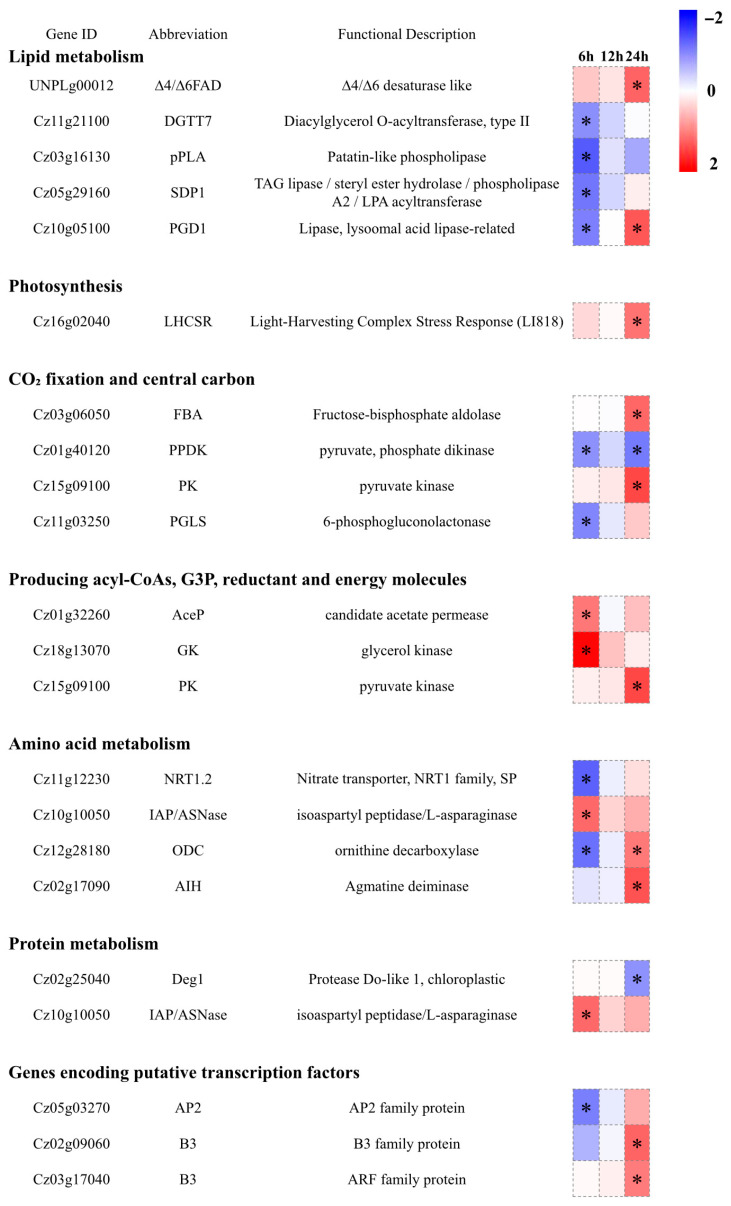
Transcriptional regulation of key metabolic pathways in response to 5-ALA treatment. Asterisks (*) indicate significantly different genes (|log_2_FC| ≥ 1 and adjusted *p* < 0.05).

We analyzed DEGs involved in lipid metabolism, carotenoid biosynthesis, photosynthesis, carbon fixation, central carbon metabolism, and the production of acyl-CoAs, glycerol-3-phosphate (G3P), reducing power, energy, and nitrogen metabolism and transcriptional regulatory pathways (Figure 6). Overall, the number of DEGs in the above pathways was relatively small (only ~20), likely because nitrogen deficiency—an effective inducer of lipid and astaxanthin accumulation—already triggers broad and substantial transcriptional reprogramming [24,36]. Consequently, the additional application of 5-ALA under nitrogen-deficient conditions did not result in further pronounced transcriptional changes. In terms of lipid metabolism, the transcript level of SDP1 (Cz05g29160) was downregulated by 2.38-fold at 6 h; SDP1 is considered to be involved in TAG degradation [50]. In contrast, PGD1 (Cz10g05100) was upregulated by 2.50-fold at 24 h; PGD1 catalyzes the degradation of nascent MGDG and releases oleic acid (C18:1n9), and is regarded as an important lipase involved in TAG synthesis [51]. These changes may collectively contribute to the increase in TAG content (Figure S1). Interestingly, diacylglycerol acyltransferase (DGTT), a rate-limiting enzyme in TAG synthesis, showed significant downregulation of DGTT7. Previous studies have shown that among the ten DGTT genes, DGTT7 maintains stable transcription under nitrogen deficiency and plays little or no role in TAG synthesis [52]. In contrast, no DEGs were identified in the carotenoid biosynthesis pathway. However, pyruvate kinase (PK), which catalyzes pyruvate formation, carries out the final step of glycolysis and serves as a key rate-limiting enzyme. The transcript level of PK (Cz15g09100) was upregulated by 2.69-fold at 24 h. This may provide more precursors (pyruvate) and ATP for lipid and carotenoid biosynthesis, thereby contributing to the increases in TFA and total carotenoids (Figure 3). In addition, glycerol kinase (GK, Cz18g13070), which catalyzes the formation of G3P from glycerol, was upregulated by 3.76-fold at 6 h, while candidate acetate permease (AceP, Cz01g32260), which facilitates acetate uptake and thus supports acyl-CoA production, was upregulated by 2.12-fold at 6 h. Together, these changes ensure sufficient carbon precursor supply for lipid biosynthesis. In addition, in amino acid and protein metabolism, the nitrate transporter NRT1.2 (Cz11g12230) was downregulated, reducing exogenous nitrogen uptake, whereas IAP/ASNase (Cz10g10050) was upregulated at 6 h, promoting endogenous nitrogen recycling. Ornithine decarboxylase (ODC) and agmatine iminohydrolase (AIH) were upregulated at 24 h, providing alternative nitrogen sources and energy through polyamine metabolism and amino acid degradation pathways, respectively. Collectively, these metabolic adjustments ensured sufficient nitrogen and energy balance, thereby supporting the substantial accumulation of lipids and astaxanthin.

**Figure 7:**
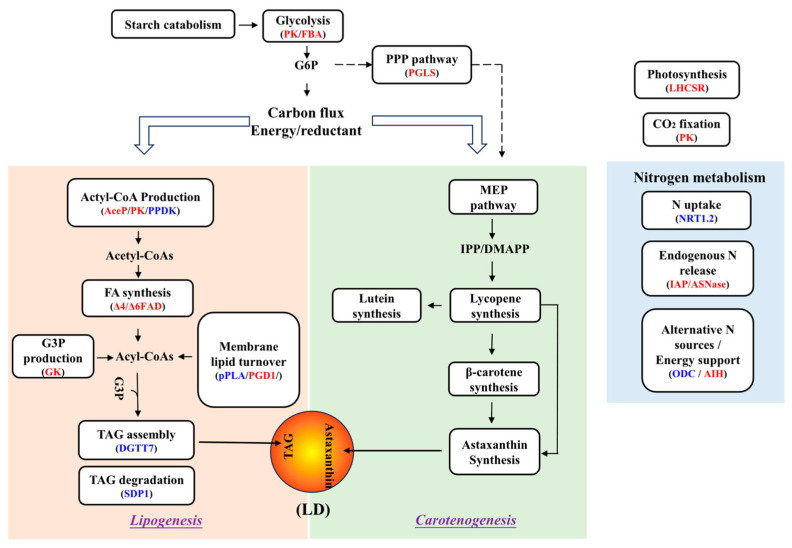
Mechanistic model of 5-ALA–induced lipogenesis and carotenogenesis in *C. zofingiensis.* Text in red, blue, and black indicate up-, down-, and non-regulated pathways, respectively. The dashed arrows indicate auxiliary (bypass) pathways.

A mechanistic model illustrating how exogenous 5-ALA regulates the metabolic pathways of *C. zofingiensis* has been proposed, as shown in Figure 7. In brief, 5-ALA enhances lipid and astaxanthin accumulation primarily by strengthening glycolysis and central carbon metabolism, thereby increasing the supply of carbon precursors (e.g., pyruvate, acetyl-CoA) and cellular energy (Figure 7). In parallel, it stimulates nitrogen metabolism to support protein remodeling and intracellular nitrogen recycling. Moreover, 5-ALA promotes the conversion of membrane lipids into TAG while simultaneously suppressing TAG degradation, collectively leading to increased TAG accumulation. At the transcriptional level, the carotenoid biosynthetic pathway remains largely stable, suggesting that the observed increases in total carotenoids and astaxanthin are primarily attributable to enhanced carbon flux rather than transcriptional upregulation of pathway genes.

### 3.6. Enhanced Productivity of Lipid and Astaxanthin Under Semi-Continuous Cultivation with 5-ALA

**Figure 8:**
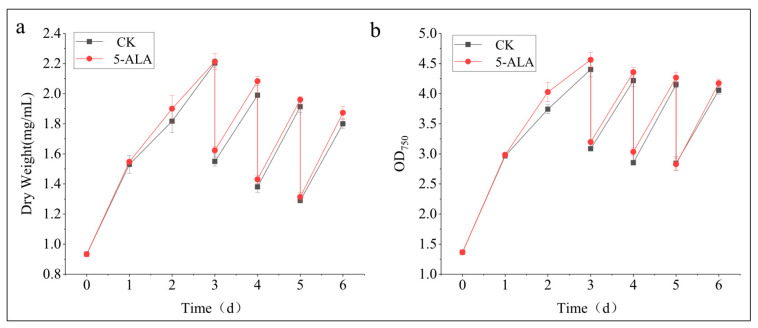
Effects of 5-ALA on biomass in *Chromochloris zofingiensis* under semi-continuous cultivation. (**a**) Dry weight; (**b**) OD_750_. Statistical significance was analyzed using one-way analysis of variance (ANOVA) followed by Dunnett’s multiple comparison test to compare treatment groups with the control group. No statistically significant differences were observed between treatments and the control (*p* > 0.05).

To evaluate the effectiveness of 5-ALA in enhancing lipid and astaxanthin production in *C. zofingiensis* under conditions more relevant to practical applications, we conducted a semi-continuous cultivation experiment. During this stage, changes in biomass are shown in Figure 8. The dry weight increased from an initial value of approximately 0.9 mg/mL to about 2.2 mg/mL on day 3, decreased to around 1.6 mg/mL after medium replacement, and subsequently recovered to approximately 1.9 mg/mL by day 6 (Figure 8a). No significant differences were observed between the treatment and control groups (*p* > 0.05). A similar trend was observed for Optical Density, with nearly overlapping curves between the two groups (Figure 8b). No significant differences were detected at any time point (*p* > 0.05). These results indicate that under nitrogen-deficient semi-continuous cultivation, 5-ALA does not significantly promote biomass accumulation. Consistent with the observations from nitrogen-deficient batch cultivation, 5-ALA fails to stimulate biomass accumulation in *C. zofingiensis*.

**Figure 9:**
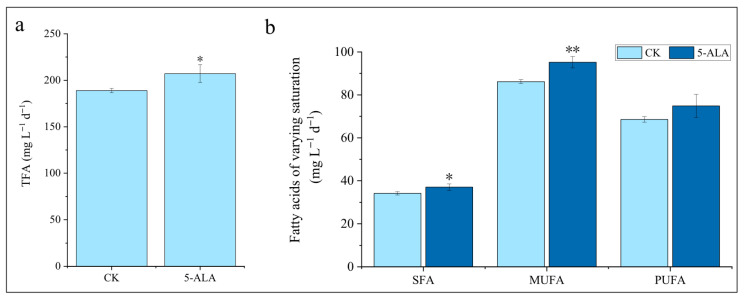
Effects of 5-ALA on lipid productivity in *Chromochloris zofingiensis*. (**a**) Total fatty acid (TFA) productivity; (**b**) TFA productivity distributed by saturation degree. Asterisks indicate significant differences compared with the control (* *p* < 0.05, ** *p* < 0.01).

**Figure 10:**
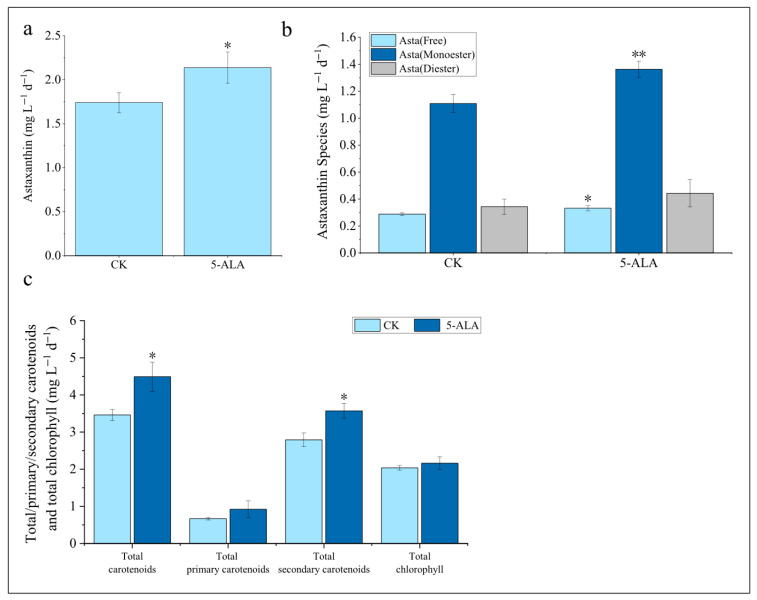
Effects of 5-ALA on pigment productivity in *Chromochloris zofingiensis*. (**a**) Total astaxanthin productivity; (**b**) Productivity of different astaxanthin species; (**c**) Productivity of total carotenoids, primary carotenoids, secondary carotenoids, and chlorophyll. Asterisks indicate significant differences compared with the control (* *p* < 0.05, ** *p* < 0.01).

Next, we investigated the effect of 5-ALA on lipid productivity under semi-continuous cultivation conditions. For TFA, the 5-ALA treatment increased lipid productivity from 188 mg L^−1^ d^−1^ to 207 mg L^−1^ d^−1^, representing a 9.6% increase (*p* < 0.05) (Figure 9a). Among the fatty acid fractions, MUFA showed the greatest increase, rising by 10.5% (*p* < 0.01) (Figure 9b, Table S3). Similarly, TAG productivity showed a more pronounced increase, rising from approximately 144 mg L^−1^ d^−1^ to 164 mg L^−1^ d^−1^, corresponding to a 13.9% increase (*p* < 0.05) (Figure S3a). Among the fatty acid fractions, MUFA showed the greatest increase, rising by 16.7% (*p* < 0.01) (Figure S3b, Table S4). In contrast, the addition of exogenous 5-ALA had no significant effect on the productivity of polar lipids (Figure S3). For pigment production, astaxanthin productivity increased from approximately 1.75 mg L^−1^ d^−1^ in the control group to 2.15 mg L^−1^ d^−1^, representing a 22.9% increase (*p* < 0.05) (Figure 10a). Among these, monoesterified astaxanthin and free astaxanthin showed the most pronounced increases, rising by 27.3% (*p* < 0.05) and 25.0% (*p* < 0.01), respectively (Figure 10b). The productivity of primary carotenoids increased by 38.1% compared with the control (*p* < 0.05), while secondary carotenoids increased by 23.3% (*p* < 0.05), with the increase mainly attributed to astaxanthin and ketolutein (Figure 10c and Figure S4). In contrast, no significant changes were observed in the productivity of total carotenoids and total chlorophyll (Figure 10c and Figure S4). Previous studies have shown that under semi-continuous cultivation of *C. zofingiensis*—particularly under nitrogen deficiency, high light, or combined nitrogen deficiency and high light—high light exerts the most effective stimulation on the simultaneous accumulation of TAG and astaxanthin, with maximum productivities reaching 173 mg L^−1^ d^−1^ and 2.01 mg L^−1^ d^−1^, respectively [53]. In comparison, the addition of a low concentration (2 μM) of 5-ALA in this study achieved comparable levels (164 mg L^−1^ d^−1^ for TAG and 2.15 mg L^−1^ d^−1^ for astaxanthin). Notably, high-light conditions are associated with substantially higher operational costs. Therefore, our results provide a potentially cost-effective strategy for promoting the simultaneous accumulation of lipids and astaxanthin in *C. zofingiensis*.

## 4. Conclusions

This study identified the optimal concentration of exogenous 5-ALA for promoting the coordinated accumulation of lipids and astaxanthin in *C. zofingiensis* and systematically elucidated its regulatory role under nitrogen-deficient conditions. At 2 μM, 5-ALA significantly enhanced lipid and astaxanthin accumulation without affecting biomass, thereby partially decoupling lipid and secondary metabolite production from growth inhibition. Mechanistically, 5-ALA promotes coordinated metabolic reprogramming by enhancing glycolysis and central carbon metabolism, increasing the supply of carbon precursors and energy, and strengthening nitrogen recycling to maintain intracellular metabolic balance. In addition, 5-ALA facilitates the conversion of membrane lipids into TAG while suppressing TAG degradation. Notably, the carotenoid biosynthesis pathway remains relatively stable at the transcriptional level, indicating that the enhanced accumulation of astaxanthin is likely driven primarily by increased metabolic flux rather than direct regulation at the gene expression level. From an application perspective, semi-continuous cultivation demonstrated that 5-ALA significantly improves lipid and astaxanthin productivity, achieving levels comparable to those obtained under high-light conditions while avoiding the high energy input associated with light intensification, thereby showing promising application potential. It should be noted that this study remains at the laboratory scale, and further scale-up cultivation and process optimization are required to evaluate the feasibility of 5-ALA for practical industrial applications. In summary, 5-ALA represents a simple and economically viable regulatory strategy for promoting the simultaneous accumulation of lipids and astaxanthin. This work provides new insights into hormone-mediated metabolic regulation and offers valuable guidance for the sustainable production of high-value microalgal bioactive compounds.

## Figures and Tables

**Table 1:** Fatty acid composition of total fatty acids (TFA) in *Chromochloris zofingiensis* under different concentrations of 5-ALA. Asterisks indicate significant differences compared with the control (* *p* < 0.05, ** *p* < 0.01, *** *p* < 0.001).

TFA (mg/g)	CK	1 μM	2 μM	4 μM	6 μM
C16:0	48.53 ± 0.46	51.64 ± 1.98	59.19 ± 0.99 ***	49.73 ± 2.56	35.38 ± 4.59 ***
C16:1 n9 △7	3.07 ± 0.48	2.99 ± 0.11	3.74 ± 0.53 *	3.12 ± 0.11	2.28 ± 0.25 *
C16:1 △3	0.68 ± 0.32	0.46 ± 0.02	0.63 ± 0.11	0.61 ± 0.09	0.54 ± 0.02
C16:2	8.23 ± 1.58	10.63 ± 0.43	9.54 ± 2.21	8.70 ± 1.87	5.54 ± 0.54
C16:3 n3	8.61 ± 1.36	7.94 ± 0.18	9.76 ± 1.57	9.02 ± 1.06	7.12 ± 0.66
C16:4	1.57 ± 0.32	1.21 ± 0.02	1.64 ± 0.57	1.63 ± 0.26	1.48 ± 0.30
C18:0	12.89 ± 2.26	10.91 ± 0.28	14.14 ± 1.72	12.70 ± 1.15	10.33 ± 1.01
C18:1 n1	129.14 ± 6.72	148.88 ± 6.28 *	148.29 ± 12.47 *	134.03 ± 13.40	89.72 ± 11.45 ***
C18:1 n7	1.50 ± 0.37	1.79 ± 0.31	1.33 ± 0.10	1.42 ± 0.24	0.92 ± 0.00 *
C18:2	57.34 ± 2.42	63.48 ± 3.53	66.47 ± 5.31 *	60.75 ± 3.45	41.48 ± 6.01 **
C18:3 n6	2.79 ± 0.26	2.20 ± 0.27	2.30 ± 0.20	2.82 ± 0.21	1.94 ± 0.15
C18:3 n3	23.87 ± 1.13	25.93 ± 0.90	27.49 ± 1.35 **	25.47 ± 1.03	18.46 ± 2.02 **
C18:4	2.13 ± 0.12	1.68 ± 0.12	1.45 ± 0.12	1.39 ± 0.10	1.66 ± 0.19

## Data Availability

The original contributions presented in this study are included in the article/Supplementary Materials. Further inquiries can be directed to the corresponding authors.

## Supplementary Materials

The following supporting information can be downloaded at: https://www.mdpi.com/article/10.3390/foods15101768/s1, Figure S1. Effects of 5-ALA on lipid metabolism in Chromochloris zofingiensis. (a) Triacylglycerol (TAG) content; (b) TAG distribution by saturation degree; (c) Polar lipid (PL) content; (d) PL distribution by saturation degree. Asterisks indicate significant differences compared with the control (* *p* < 0.05, ** *p* < 0.01, *** *p* < 0.001); Figure S2. Pigment profiling of Chromochloris zofingiensis in response to 5-ALA. Asterisks indicate significant differences compared with the control (* *p* < 0.05, ** *p* < 0.01, *** *p* < 0.001); Figure S3. Effects of 5-ALA on lipid productivity in Chromochloris zofingiensis under a semi-continuous cultivation mode. (a) Triacylglycerol (TAG) productivity; (b) TAG productivity distributed by saturation degree; (c) Polar lipid (PL) productivity; (d) PL productivity distributed by saturation degree. Asterisks indicate significant differences compared with the control (* *p* < 0.05, ** *p* < 0.01); Figure S4. Effects of 5-ALA on individual pigment productivity in Chromochloris zofingiensis under a semi-continuous cultivation mode. Asterisks indicate significant differences compared with the control (* *p* < 0.05); Table S1. Fatty acid composition of triacylglycerol (TAG) in Chromochloris zofingiensis under different concentrations of 5-ALA. Asterisks indicate significant differences compared with the control (* *p* < 0.05, ** *p* < 0.01, *** *p* < 0.001); Table S2. Fatty acid composition of phospholipids (PL) in Chromochloris zofingiensis under different concentrations of 5-ALA. Asterisks indicate significant differences compared with the control (* *p* < 0.05); Table S3. Productivity of different types of fatty acids in total fatty acids (TFA) of C. zofingiensis treated with 5-ALA under a semi-continuous cultivation mode. Asterisks indicate significant differences compared with the control (* *p* < 0.05, ** *p* < 0.01); Table S4. Productivity of different types of fatty acids in triacylglycerol (TAG) of C. zofingiensis treated with 5-ALA under a semi-continuous cultivation mode. Asterisks indicate significant differences compared with the control (* *p* < 0.05); Table S5. Productivity of different types of fatty acids in phospholipids (PL) of C. zofingiensis treated with 5-ALA under a semi-continuous cultivation mode. Asterisks indicate significant differences compared with the control (* *p* < 0.05).
