# Supplementary material for: 5-Aminolevulinic Acid Drives Coordinated Astaxanthin and Lipid Accumulation in Green Alga Chromochloris zofingiensis
Source: Foods. 2026 May 17;15(10):1768. doi: 10.3390/foods15101768 (PMC13206523; doi:10.3390/foods15101768)
Supplement: Supplementary file 1 [file foods-15-01768-s001.zip › foods-4314770-supplementary.pdf]

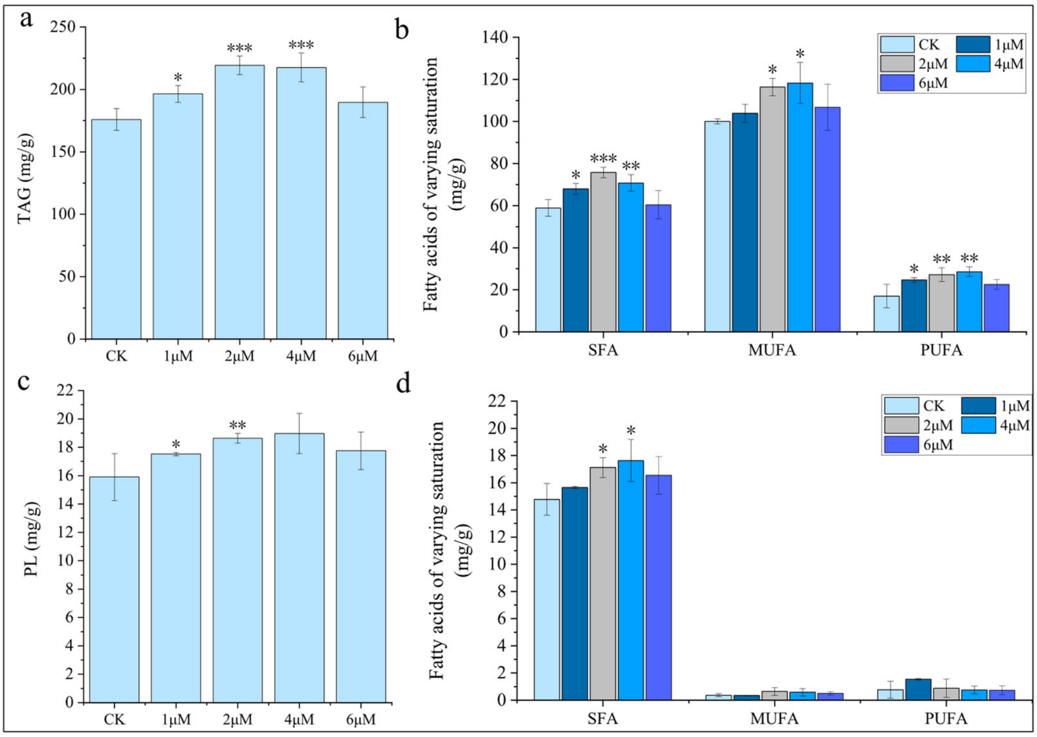

Figure S1. Effects of 5-ALA on lipid metabolism in *Chromochloris zofingiensis*. (a) Triacylglycerol (TAG) content; (b) TAG distribution by saturation degree; (c) Polar lipid (PL) content; (d) PL distribution by saturation degree. Asterisks indicate significant differences compared with the control (*$p < 0.05$, **$p < 0.01$, ***$p < 0.001$).

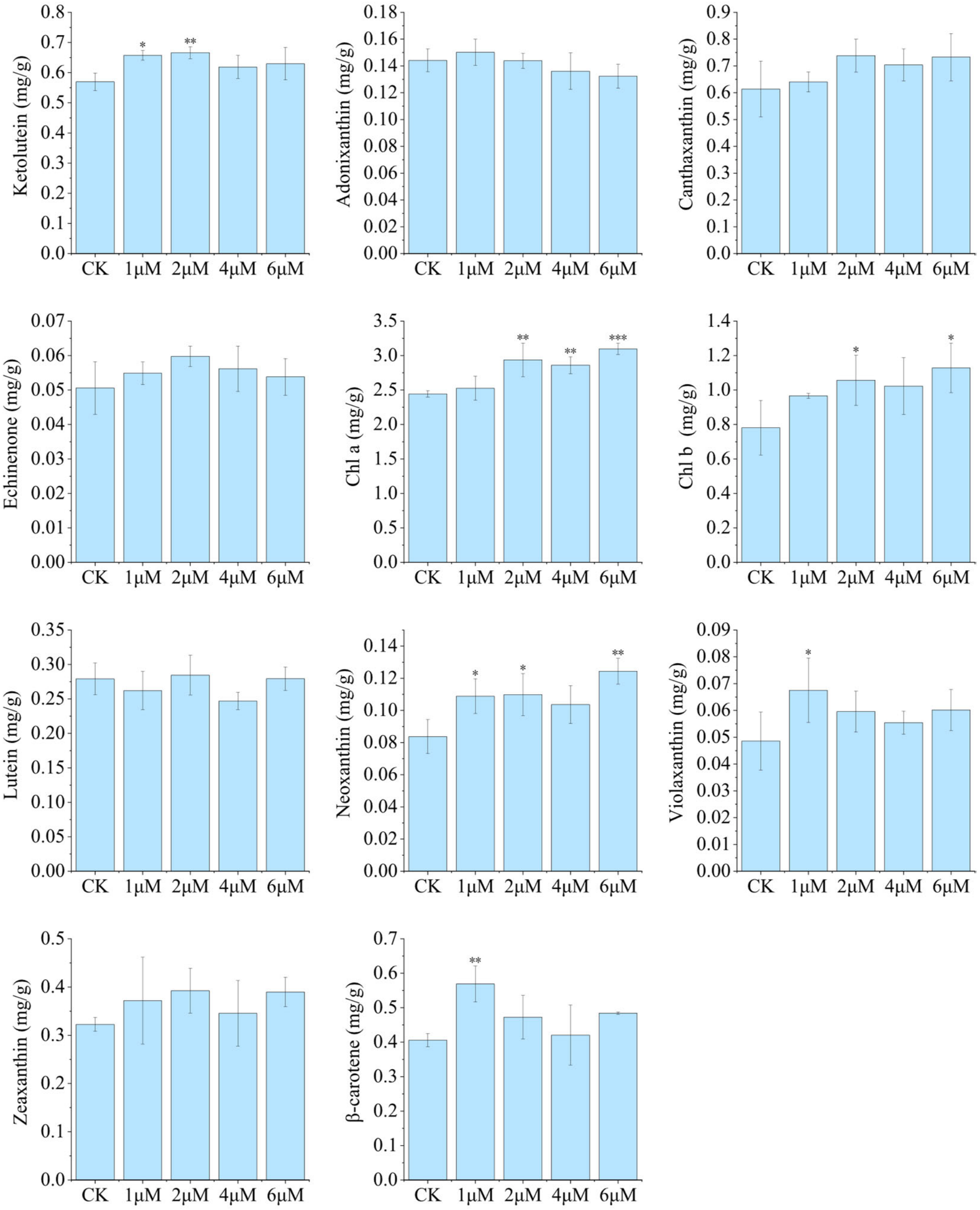

Figure S2. Pigment profiling of *Chromochloris zofingiensis* in response to 5-ALA. Asterisks indicate significant differences compared with the control (*$p < 0.05$, **$p < 0.01$, ***$p < 0.001$).

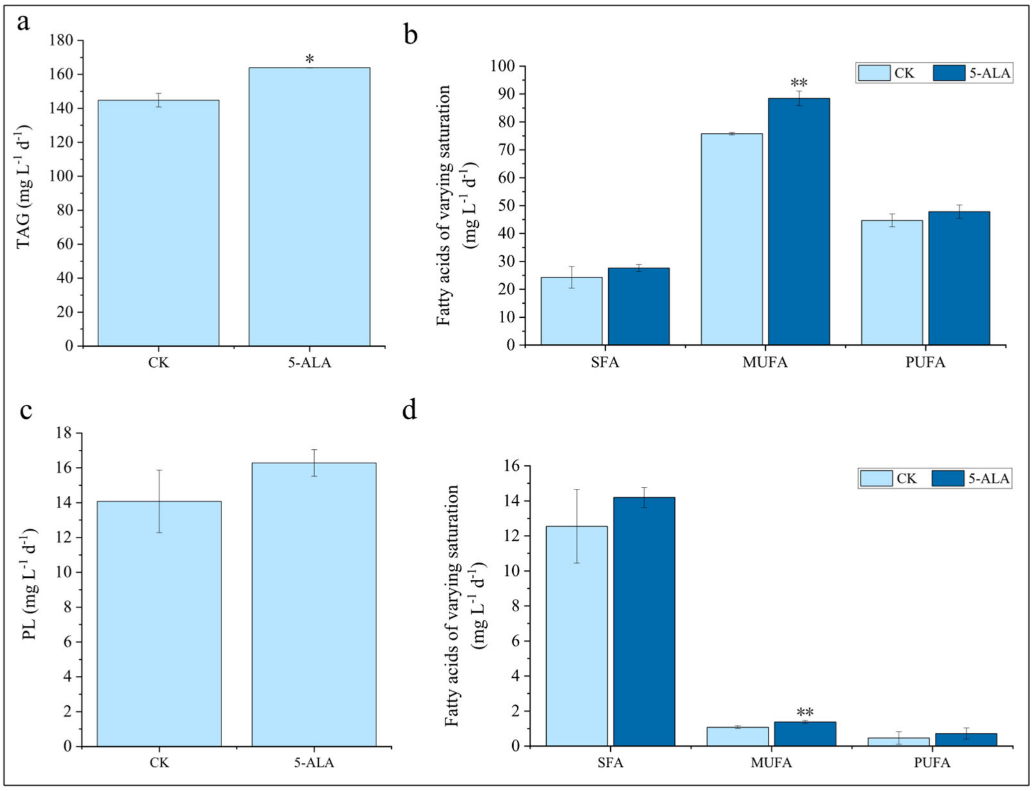

Figure S3. Effects of 5-ALA on lipid productivity in *Chromochloris zofingiensis* under a semi-continuous cultivation mode. (a) Triacylglycerol (TAG) productivity; (b) TAG productivity distributed by saturation degree; (c) Polar lipid (PL) productivity; (d) PL productivity distributed by saturation degree. Asterisks indicate significant differences compared with the control (*$p < 0.05$, **$p < 0.01$).

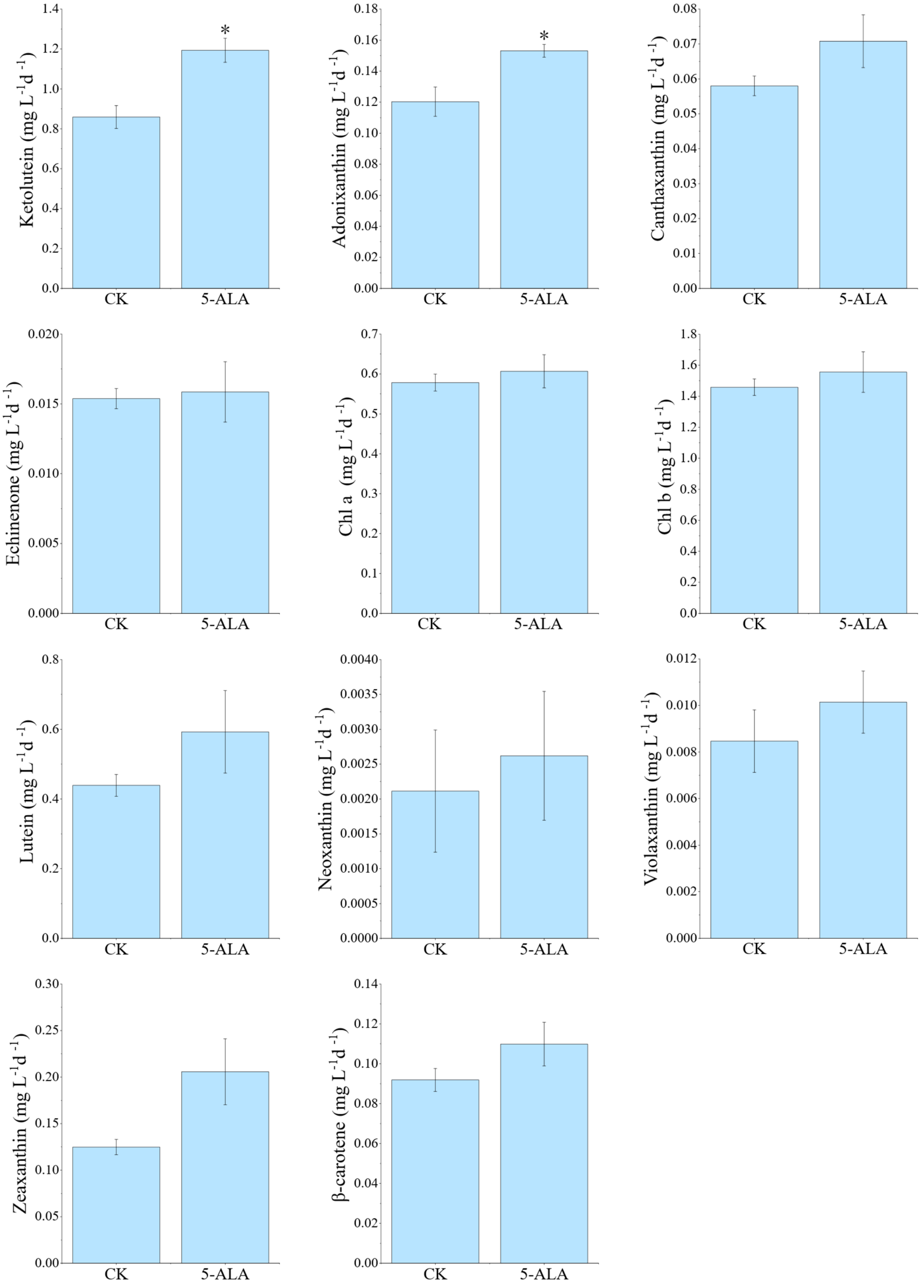

Figure S4. Effects of 5-ALA on individual pigment productivity in *Chromochloris zofingiensis* under a semi-continuous cultivation mode. Asterisks indicate significant differences compared with the control (*$p < 0.05$).

Table S1. Fatty acid composition of triacylglycerol (TAG) in *Chromochloris zofingiensis* under different concentrations of 5-ALA. Asterisks indicate significant differences compared with the control (*p < 0.05, **p < 0.01, ***p < 0.001).

TAG（mg/g）	CK	1μM	2μM	4μM	6μM
C16:0	34.21 ± 4.28	41.65 ± 1.45*	47.10 ± 2.91**	42.74 ± 2.43*	34.16 ± 5.16
C16:1 Δ7 n9	2.24 ± 0.22	2.20 ± 0.08	2.58 ± 0.26	2.48 ± 0.05	2.25 ± 0.25
C16:1 Δ3t	0.38 ± 0.15	0.21 ± 0.01	0.41 ± 0.16	0.42 ± 0.18	0.44 ± 0.21
C16:2	1.64 ± 0.95	2.94 ± 0.12	2.81 ± 0.50	2.91 ± 0.37	1.79 ± 1.40
C16:3 n3	0.70 ± 0.45	1.30 ± 0.03*	1.42 ± 0.18**	1.53 ± 0.20**	1.23 ± 0.05*
C18:0	24.69 ± 0.49	26.29 ± 1.68	28.67 ± 2.71*	28.01 ± 1.81*	26.23 ± 1.68
C18:1 n9	97.40 ± 0.88	101.45 ± 4.18	113.37 ± 3.86*	115.37 ± 9.57*	104.09 ± 10.64
C18:2	11.02 ± 4.40	16.97 ± 0.94*	19.45 ± 2.41**	20.41 ± 1.88***	16.54 ± 0.66*
C18:3 n3	3.64 ± 0.24	3.47 ± 0.12	3.51 ± 0.61	3.72 ± 0.54	2.97 ± 0.50

Table S2. Fatty acid composition of phospholipids (PL) in *Chromochloris zofingiensis* under different concentrations of 5-ALA. Asterisks indicate significant differences compared with the control (*p < 0.05).

PL（mg/g）	CK	1μM	2μM	4μM	6μM
C16:0	13.84 ± 1.21	14.90 ± 0.07	16.07 ± 0.61*	16.49 ± 1.32*	15.61 ± 1.42
C16:1 Δ7 n9	0.07 ± 0.02	0.05 ± 0.00	0.21 ± 0.22	0.20 ± 0.19	0.07 ± 0.02
C16:1 Δ3t	0.24 ± 0.09	0.21 ± 0.01	0.30 ± 0.11	0.26 ± 0.08	0.34 ± 0.09
C16:2	0.12 ± 0.15	0.31 ± 0.01	0.15 ± 0.18	0.15 ± 0.18	0.14 ± 0.15
C16:3 n3	0.05 ± 0.03	0.09 ± 0.00	0.05 ± 0.04	0.05 ± 0.04	0.06 ± 0.03
C18:0	0.93 ± 0.20	0.75 ± 0.02	1.05 ± 0.27	1.15 ± 0.28	0.93 ± 0.15
C18:1 n9	0.06 ± 0.01	0.08 ± 0.01	0.13 ± 0.11	0.13 ± 0.11	0.07 ± 0.01
C18:2	0.30 ± 0.35	0.74 ± 0.04*	0.35 ± 0.37	0.16 ± 0.02	0.18 ± 0.02
18:3 n6	0.06 ± 0.07	0.01 ± 0.00	0.04 ± 0.03	0.12 ± 0.10	0.04 ± 0.02
C18:3 n3	0.23 ± 0.12	0.38 ± 0.01	0.28 ± 0.11	0.27 ± 0.13	0.31 ± 0.15

Table S3. Productivity of different types of fatty acids in total fatty acids (TFA) of *C. zofingiensis* treated with 5-ALA under a semi-continuous cultivation mode. Asterisks indicate significant differences compared with the control (*p < 0.05, **p < 0.01).

TFA (mg/L/d)	CK	5-ALA
C16:0	28.26 ± 0.77	30.86 ± 1.41*
C16:1 n9 △7	1.81 ± 0.31	2.25 ± 0.10**
C16:1 △3	0.25 ± 0.00	0.27 ± 0.01
C16:2	3.92 ± 0.24	4.16 ± 0.28
C16:3 n3	8.08 ± 0.23	8.76 ± 0.63
C16:4	1.24 ± 0.02	1.40 ± 0.11
C18:0	5.93 ± 0.09	6.22 ± 0.62
C18:1 n1	83.58 ± 0.77	92.09 ± 2.53*
C18:1 n7	0.54 ± 0.09	0.61 ± 0.09
C18:2	37.08 ± 1.71	39.41 ± 3.00
C18:3 n6	0.91 ± 0.01	1.00 ± 0.07
C18:3 n3	17.57 ± 0.45	18.90 ± 1.24
C18:4	1.12 ± 0.04	1.23 ± 0.09

Table S4. Productivity of different types of fatty acids in triacylglycerol (TAG) of *C. zofingiensis* treated with 5-ALA under a semi-continuous cultivation mode. Asterisks indicate significant differences compared with the control (*p < 0.05).

TAG (mg/L/d)	CK	5-ALA
C16:0	20.48 ± 3.45	23.10 ± 1.61
C16:1 n9 △7	0.60 ± 0.11	0.93 ± 0.08*
C16:1 △3	0.24 ± 0.14	0.81 ± 0.60
C16:2	1.55 ± 0.13	1.35 ± 0.87
C16:3 n3	2.24 ± 1.69	2.42 ± 1.96
C16:4	0.72 ± 0.15	0.88 ± 0.06
C18:0	3.83 ± 0.41	4.55 ± 0.42
C18:1 n1	74.96 ± 0.51	86.72 ± 3.09*
C18:2	30.22 ± 0.14	30.39 ± 0.05
C18:3 n6	0.63 ± 0.05	0.87 ± 0.09*
C18:3 n3	8.52 ± 0.46	11.27 ± 1.27*
C18:4	0.82 ± 0.11	0.66 ± 0.29

Table S5. Productivity of different types of fatty acids in phospholipids (PL) of *C. zofingiensis* treated with 5-ALA under a semi-continuous cultivation mode. Asterisks indicate significant differences compared with the control (*p < 0.05).

PL(mg/L/d)	CK	5-ALA
C16:0	12.40 ± 2.08	14.02 ± 0.59
C16:1 n9 △7	0.03 ± 0.01	0.04 ± 0.01
C16:1 △3	0.01 ± 0.01	0.02 ± 0.03
C16:2	0.03 ± 0.03	0.04 ± 0.01
C16:3 n3	0.05 ± 0.01	0.05 ± 0.01
C16:4	0.01 ± 0.00	0.01 ± 0.00
C18:0	0.15 ± 0.04	0.18 ± 0.02
C18:1 n1	1.03 ± 0.08	1.32 ± 0.10*
C18:2	0.30 ± 0.26	0.48 ± 0.21
C18:3 n6	0.01 ± 0.00	0.01 ± 0.00
C18:3 n3	0.07 ± 0.06	0.13 ± 0.08
